# Ownership and use of insecticide-treated nets in Myanmar: insights from a nationally representative demographic and health survey

**DOI:** 10.1186/s12936-024-04994-z

**Published:** 2024-05-29

**Authors:** Kyawt Mon Win, Kyaw Lwin Show, Jetsumon Sattabongkot, Pyae Linn Aung

**Affiliations:** 1 Mahidol Vivax Research Unit, Faculty of Tropical Medicine, Mahidol University, Bangkok, Thailand; 2 Independent Researcher, Yangon, Myanmar

**Keywords:** Factors, Insecticide-treated net, Malaria, Myanmar, Ownership, Use

## Abstract

**Background:**

Malaria poses a substantial public health threat in Myanmar, indicating the need for rigorous efforts to achieve elimination of the disease nationwide by 2030. The use of insecticide-treated nets (ITNs) forms part of a pivotal strategy for preventing transmission. This study explored the ownership and use of ITNs in Myanmar and identified factors associated with non-use of ITNs.

**Methods:**

Household datasets from the 2015–2016 Myanmar Demographic and Health Survey were utilised, which encompassed all household members except children under the age of five. Descriptive statistics and inferential tests, including simple and multiple logistics regression models and Pearson correlations, were employed for analysis. All analyses, taking the two-stage stratified cluster sampling design into account, used weighting factors and the “*svyset”* command in STATA. The ownership and use of bed nets were also visualised in QGIS maps.

**Results:**

Among the 46,507 participants, 22.3% (95% CI 20.0%, 24.5%) had access to ITNs, with only 15.3% (95% CI 13.7, 17.1%) sleeping under an ITN the night before the survey. Factors associated with the non-use of ITNs included age category (15–34 years—aOR: 1.17, 95% CI 1.01, 1.30; 50+ years—aOR: 1.19, 95% CI 1.06, 1.33), location (delta or lowland—aOR: 5.39, 95% CI 3.94, 7.38; hills—aOR: 1.80, 95% CI 1.20, 2.71; plains—aOR: 3.89, 95% CI 2.51, 6.03), urban residency (aOR: 1.63, 95% CI 1.22, 2.17), and wealth quintile (third—aOR: 1.38, 95% CI 1.08, 1.75; fourth—aOR: 1.65, 95% CI 1.23, 2.23; fifth—aOR: 1.47, 95% CI 1.02, 2.13). A coherent distribution of the ownership and use of ITNs was seen across all states/regions, and a strong correlation existed between the ownership and use of ITNs (r: 0.9795, 95% CI 0.9377, 0.9933, alpha < 0.001).

**Conclusions:**

This study identified relatively low percentages of ITN ownership and use, indicating the need to increase the distribution of ITNs to achieve the target of at least one ITN per every two people. Strengthening the use of ITNs requires targeted health promotion interventions, especially among relatively affluent individuals residing in delta or lowland areas, hills, and plains.

## Background

The countries of the Greater Mekong Subregion have set the ambitious target of eliminating malaria in their territories by 2030 [1]. However, malaria continues to pose a significant public health threat in certain nations and Myanmar in particular [2, 3]. According to the latest World Malaria Report of the World Health Organization (WHO) [1], Myanmar had 157,533 cases of malaria in 2022, reflecting a near doubling from the cases reported in 2021. Alarmingly, malaria-associated mortality also doubled, with 20 deaths reported in 2022, highlighting the urgent need for intensified control measures. Across the 330 townships in the country, almost 90%—excluding some urbanized areas in central Myanmar, such as downtown Yangon, Mandalay, and Bago—have either active or residually active foci, with the presence of major malaria vectors [4, 5]. *Anopheles* mosquitoes, particularly *Anopheles minimus* and *Anopheles dirus*, have been identified as major malaria vectors that are distributed widely across the nation [6, 7]. To break the cycle of active transmission and reduce the disease burden, effective control measures, including early diagnosis, prompt treatment, vector control, and prevention of mosquito bites, are imperative.

In Myanmar, the National Malaria Control Programme (NMCP), along with local and international non-governmental organisations, under the guidance of the Department of Public Health, primarily focus on two key activities for vector control: the distribution of insecticide-treated nets (ITNs) and indoor residual spraying (IRS) [4]. IRS interventions are typically implemented in specific circumstances, such as in the face of unusual increases in malaria incidence or in areas that feature exceptionally high malaria prevalence [5, 8]. ITNs, bed nets impregnated with insecticides, come in two forms: self-impregnated nets for short-term use and long-lasting insecticidal nets (LLINs) for long-term use [9]. ITNs are distributed biennially with additional annual topping up, targeting high-risk groups such as migrant workers, pregnant women, and young children [4, 5]. Following WHO recommendations, the distribution of nets is intended to ensure that every two individuals in areas at high risk of malaria possess at least one ITN [10]. In 2022, over 550,000 ITNs were distributed across Myanmar [1]. While people living in malaria-free areas typically do not receive ITNs, ITN ownership should be ideally be high in malaria hotspots. However, studies in Myanmar and Thailand suggest that ownership status may not always directly correlate with actual ITN usage [11–13].

Malaria prevention is paramount to reduce unnecessary mortality, break the transmission chain, and prevent further transmission. The use of ITNs is a recommended preventive measure [9, 14]. However, the use of ITN remains suboptimal, particularly in relation to specific populations in Myanmar: 9.0%–13.0% in migrant workers [15], 15.9% in pregnant women [16], and 44.0% in children under 5 years old [17]. Several challenges hinder ITN use, including inadequate ownership rates for ITNs and difficulty setting up nets at workplaces, particularly for those who work during night-time hours [12, 13, 18, 19]. Incorrect beliefs regarding ITNs also contribute to low rates of usage, where some individuals rejecting ITN use to maintain privacy, in protect against cold weather, and in fear of potential allergies or other negative reactions to the insecticide in the nets [13, 18, 20, 21]. The misconceptions at the bottom of these negative attitudes can significantly impact ITN utilisation. In addition, the individual characteristics of age, sex, and socioeconomic status have crucial roles to play in influencing the appropriate use of ITNs [12, 19, 22]. To address these challenges and enhance effective ITN use, interventions must be tailored to the specific needs of the target population. It is essential to identify the most effective health promotion strategies for specific demographics to ensure the widespread adoption and consistent use of ITNs.

Nationally representative surveys addressing malaria-related issues are infrequent in Myanmar, mainly due to constrained human resources and budgets. The last survey of malaria indicators survey took place in 2015, and its final report and detailed data have not yet been released [23]. As a result, the primary source of nationally representative data available is the Myanmar Demographic Health Survey 2015–2016 (MDHS 2015–2016) [24]. The most recent MDHS report, published in 2017, touched briefly on malaria-related topics, including the use of ITNs and health-seeking behaviours that are related to malaria. Raw datasets from the MDHS are available for analysis. A PubMed search identified two publications related to MDHS that targeted specific population groups. The first focused the carers of children under 5 years of age, reporting overall ownership and use of ITNs and factors such as location and wealth status that influenced non-use [25]. The other publication examined ITN use among pregnant women, identifying the area of residence as a factor that is associated with non-use [16]. However, there has been no comprehensive exploration of ITN ownership and use in the general population. Given this, this study seeks to determine the overall ownership and use of bed nets, focusing in particular on the factors that are associated with the non-use of ITNs, utilising secondary data from the MDHS 2015–2016.

## Methods

### Study design and data sources

This study constitutes a secondary analysis of data that were derived from the MDHS 2015–2016 [24].

### Myanmar demographic and health survey

The MDHS 2015–2016 was the first and most recent national-level survey conducted under the auspices of the Ministry of Health (MoH) in Myanmar. The sampling framework of the survey and its samples were drawn from the most recent population census of the country, from 2014. Employing a two-stage sampling design encompassing both urban and rural areas across all seven states, eight regions, and one union territory in Myanmar, 442 clusters were selected in the first stage. An average of 30 households from each cluster, for 13,260 households, were systematically sampled in the second stage. All women aged 15–49 years in each selected household and men aged 15–49 years in every second household were included in the survey. In addition, visitors who had slept in the given household the night before the survey were eligible to participate.

This survey employed three sets of validated questionnaires, covering household, male, and female participants, addressing country-specific contexts, basic demographic information, socioeconomic factors, and health issues. The questionnaires underwent initial validation in English, followed by translation into Burmese through a back-translation process. The data collection training for hundreds of data assistants from health departments and civil society organisations across the country was conducted by nine master trainers from the MoH. Data collection occurred between December 2015 and July 2016, utilising tablet computers, and implementing data validation at various levels.

For this study, a dataset with raw data from the household questionnaire, the Household Member Recode (PR), files within the DHS datasets, was requested and used in the analysis. In the household questionnaire, each respondent represented their household and provided responses that covered themselves and all other members, including young children, older individuals, and visitors who were residing in the same household at the time of the survey. A previous publication [25] addressed ITN usage among children under 5 years old utilizing the same dataset, but this study included all household members except those in this age group, totalling 46,507 participants for the present analysis.

### Variables

Net ownership was categorised as “yes” if the household possessed any type of mosquito bed net for sleeping. Access to an ITN was determined by the de facto household population that was eligible to sleep under an ITN, taking into account that each ITN in the household could be used by up to two people. “Slept under an ITN” was categorised as “yes” if the household member had slept under an ITN the night before the survey. The independent variables included household members’ age, level of education, region, place of residence, wealth quintile, number of household members, sex of the head of household, and access to mass media. Age was stratified into five groups: < 5, 5 to 14, 15 to 34, 35 to 49, and 50+ years old. Regions were categorised based on their characteristics: delta and lowland (Ayeyarwady, Yangon, and Bago Regions, Mon, and the Karen States), hilly (Kachin, Kayah, Chin, and the Shan States), coastal (Rakhine State and Tanintharyi Region), and plains (Magway, Mandalay, Sagaing Regions, and Nay Pyi Taw Union Territory) regions. Household size was classified into three groups: one to four, five to eight, and more than eight members. Access to mass media was recorded as “yes” if the household owned either a radio or a television.

### Data handling and analysis

This study employed a household dataset derived from the MDHS for comprehensive analysis. This analytical approach incorporated multiple stages. First, overall proportions were calculated for the ownership of any net and ITN, as well as the use of bed nets the night before the survey for either any net or an ITN. To present these proportions visually, bar graphs were generated, representing the 95% confidence intervals (95% CI) for each value as well. Second, a descriptive analysis of the general characteristics of study participants was conducted, including numerical values and percentages. The study likewise explored the associations between independent variables and the non-use of ITN through both simple and multiple logistic regression models. This presentation included crude odds ratios and adjusted odds ratios (aORs) with their corresponding 95% CIs. All of the variables from the simple regression model were retained in the adjusted model to establish genuine associations between the independent and dependent variables, setting aside their significance. In addition, this study investigated the potential correlations between the ownership and use of bed nets using Pearson correlation analysis, providing coefficients (r) with their respective 95% CIs and alpha values. All of the statistical analyses were performed using STATA (version 15, STATA Corp., College Station, TX, USA), taking the two-stage stratified cluster sampling design with the application of weight factors and the command *svyset*. Finally, QGIS (version 3.34) was employed to create the spatial visualisation, taking into account the distribution of ownership and the use of any net or ITN across all states/regions in Myanmar.

## Results

### Ownership and use of bed nets

**Fig. 1:**
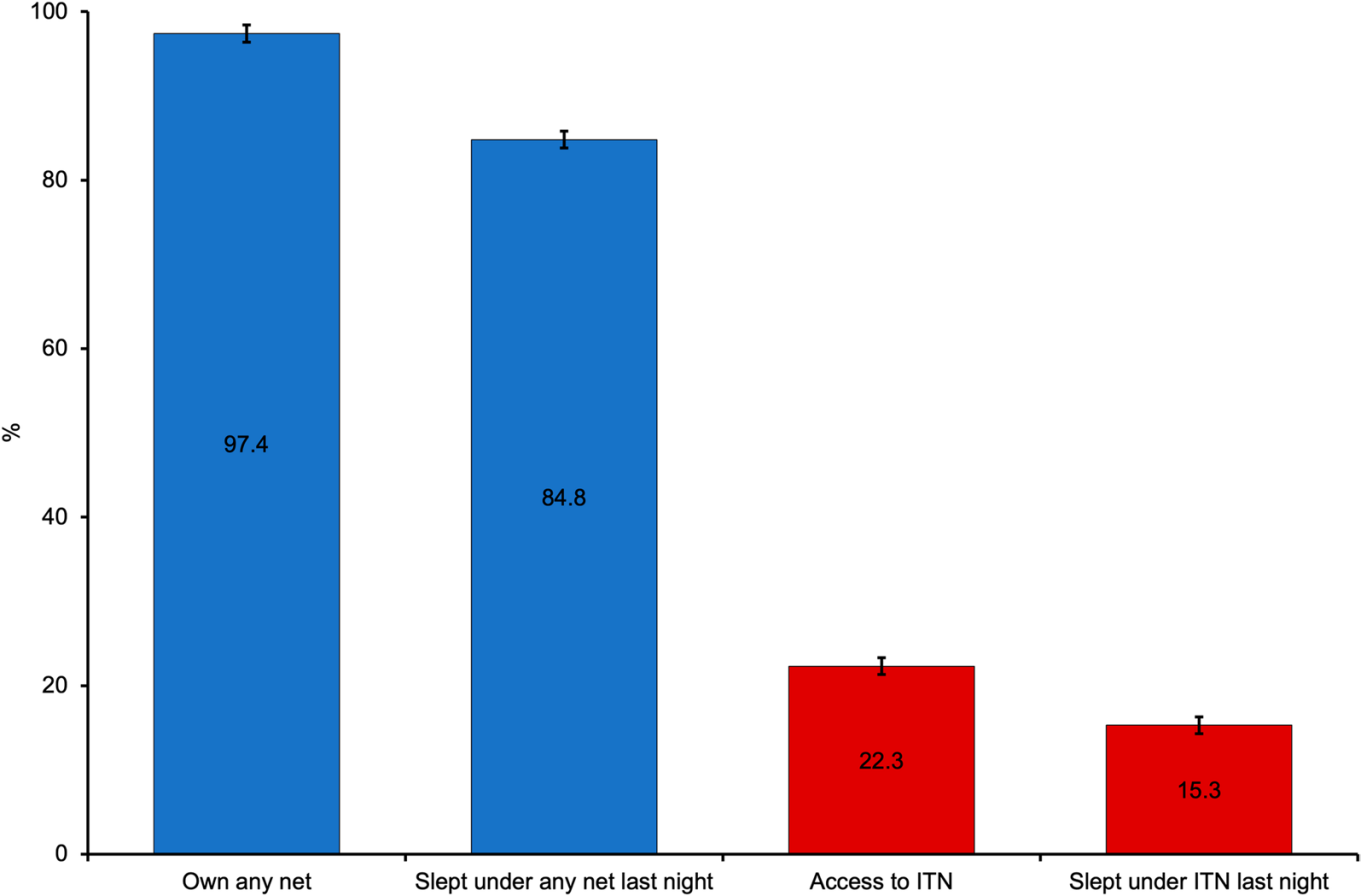
Ownership and use of bed nets

Among the 46,507 study participants, nearly all (97.4%, 95% CI 96.4%, 98.1%) possessed some form of net, be it a conventional net or an ITN. Of these, the majority (84.8%, 95% CI 83.3%, 86.2%) reported sleeping under a net the night before the survey. However, only one-fifth (22.3%, 95% CI 20.0%, 24.5%) had access to an ITN. A relatively small proportion (15.3%, 95% CI 13.7%, 17.1%) reported sleeping under an ITN on the night before the survey (Fig. 1).

### Characteristics of the study participants

Of the total sample of 46,507 study participants, nearly one-third were aged 15 to 34 years (31.7%). Additionally, more than two-fifths of participants (41.8%) had completed primary education level. The majority of participants (72.2%) resided in rural areas, 43.1% were from delta and lowland regions, 72.4% had access to mass media through a radio or television, and 80.4% lived in households headed by males. Half of the participants (49.5%) reported having five to eight household members (Table 1).

**Table 1:** Background characteristics of the household population (n = 46,507)

Characteristics	Number	Percentage
Age in years		
5–14	10,413	(22.4)
15–34	14,731	(31.7)
35–49	9992	(21.5)
50+	11,366	(24.4)
Don’t know/missing	5	(0.01)
Education		
No education	9639	(20.7)
Primary	19,435	(41.8)
Secondary or higher	17,395	(37.4)
Don’t know/missing	38	(0.1)
Region		
Delta and lowland	20,063	(43.1)
Hills	7148	(15.4)
Coastal	3993	(8.6)
Plains	15,303	(32.9)
Residence		
Urban	12,928	(27.8)
Rural	33,579	(72.2)
Wealth quintiles		
First (poorest)	8690	(18.7)
Second	9129	(19.6)
Third	9417	(20.3)
Fourth	9554	(20.5)
Fifth (richest)	9717	(20.9)
Number of household members		
1–4	19,534	(42.0)
5–8	23,006	(49.5)
> 8	3967	(8.5)
Sex of household head		
Male	37,401	(80.4)
Female	9106	(19.6)
Access to mass media (radio or television)		
Yes	33,693	(72.4)
No	12,814	(27.6)

### Association between the general characteristics of the study participants and non-use of insecticide-treated nets

A significant proportion of the study participants (84.7%, n = 39,377, 95% CI 82.90%, 86.29%) did not sleep under an ITN on the night before the survey. In particular, individuals aged over 50 years (86.0%), those with a secondary or higher education level (87.0%), residents of delta and lowland areas (90.6%), individuals from urban regions (90.7%), those in the highest wealth quintile (89.4%), households with one to four members (86.2%), families led by a female head of household (85.3%), and households with access to mass media, including radio and television (86.3%), were more inclined not to use ITNs on the final night before the survey (Table 2).

**Table 2:** Factors associated with non-use of insecticide-treated nets among the household population (n = 46,507)

Characteristics	ITN non-use		cOR	95% CI	aOR	95% CI
	n	(%)				
Age in years						
5–14	8530	(81.9)	Ref		Ref	
15–34	12,566	(85.3)	1.28	1.17, 1.41	1.17	1.01, 1.30
35–49	8498	(85.1)	1.26	1.14, 1.38	1.05	0.96, 1.15
50+	9779	(86.0)	1.36	1.22, 1.52	1.19	1.06, 1.33
Don’t know/missing	5	(100)	–		–	
Education						
No education	7757	(80.5)	0.62	0.49, 0.77	0.97	0.78, 1.19
Primary	16,453	(84.7)	0.83	0.73, 0.93	1.09	0.98, 1.22
Secondary or higher	15,132	(87.0)	Ref		Ref	
Don’t know/missing	36	(93.2)	–		–	
Region						
Delta and lowland	18,177	(90.6)	6.31	4.59, 8.69	5.39	3.94, 7.38
Hills	5433	(76.0)	2.07	1.38, 3.12	1.80	1.20, 2.71
Coastal	2413	(60.4)	Ref		Ref	
Plains	13,355	(87.3)	4.49	2.93, 6.88	3.89	2.51, 6.03
Residence						
Urban	11,727	(90.7)	2.09	1.59, 2.76	1.63	1.22, 2.17
Rural	27,651	(82.3)	Ref		Ref	
Wealth quintiles						
First (poorest)	6747	(77.6)	Ref		Ref	
Second	7469	(81.8)	1.30	1.09, 1.54	1.15	0.97, 1.36
Third	8044	(85.4)	1.69	1.33, 2.14	1.38	1.08, 1.75
Fourth	8429	(88.2)	2.16	1.64, 2.83	1.65	1.23, 2.23
Fifth (richest)	8689	(89.4)	2.43	1.79, 3.31	1.47	1.02, 2.13
Number of household members						
1–4	16,841	(86.2)	Ref		Ref	
5–8	19,207	(83.5)	0.81	0.70, 0.93	0.88	0.76, 1.02
> 8	3330	(83.9)	0.84	0.62, 1.12	0.95	0.71, 1.28
Sex of household head						
Male	31,614	(84.5)	Ref		Ref	
Female	7764	(85.3)	1.06	0.91, 1.23	0.96	0.83, 1.10
Access to mass media (radio or television)						
Yes	29,078	(86.3)	Ref		Ref	
No	10,299	(80.4)	0.65	0.54, 0.78	1.07	0.91, 1.24

ITN: insecticide-treated net; cOR: crude odds ratio; aOR: adjusted odds ratio; 95% CI: 95% confidence interval

Multiple logistic regression analyses showed that older age participants (50+ years) had higher odds of not using ITNs than children aged 5–14 years (aOR: 1.19, 95% CI 1.06, 1.33). Furthermore, individuals living in delta and lowland regions had significantly greater odds of not using ITNs than those residing in coastal regions (aOR: 5.39, 95% CI 3.94, 7.38). Likewise, individuals in hilly regions (aOR: 1.80, 95% CI 1.20, 2.71) and plains (aOR: 3.89, 95% CI 2.51, 6.03) exhibited greater odds of not using ITNs. Urban residents were less likely to use ITNs than individuals from rural areas (aOR: 1.63, 95% CI 1.22, 2.17). In addition, participants in higher wealth quintiles demonstrated a higher likelihood of non-using ITNs than individuals in the poorest wealth quintile (third quintile: aOR: 1.38, 95% CI 1.08, 1.75; fourth quintile: aOR: 1.65, 95% CI 1.23, 2.23; fifth quintile: aOR: 1.47, 95% CI 1.02, 2.13). All other independent variables, namely, education level, size of household, sex of the head of household, and access to mass media, were not statistically associated with non-use of ITNs in the present study (Table 2).

### Spatial distribution of ownership and use of bed nets

**Fig. 2:**
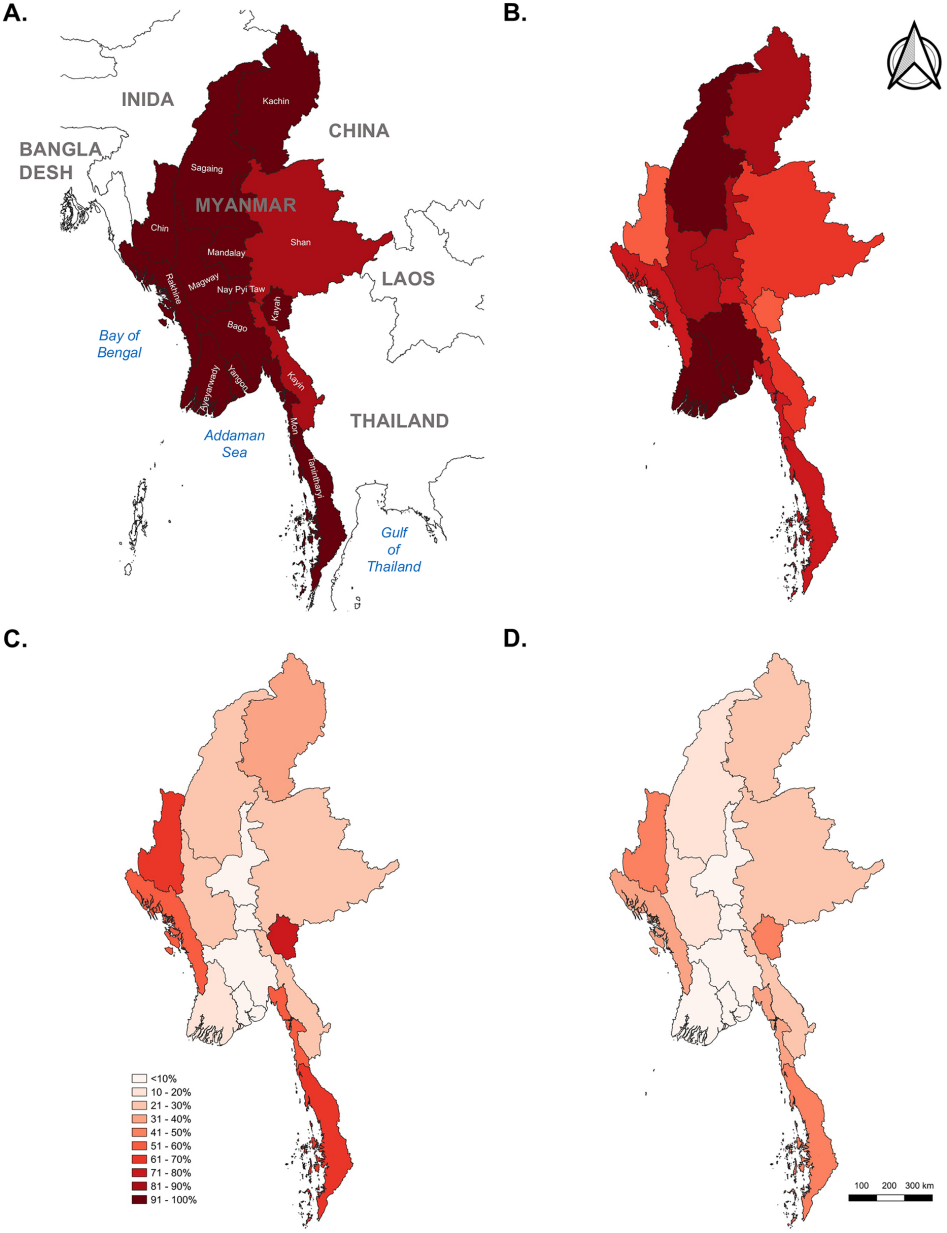
Spatial distribution of ownership and use of bed nets at state/regional level (**A** Any net ownership; **B** Slept under any net last night; **C** Access to insecticide-treated net; **D** Slept under insecticide-treated net last night)

Figure 2 presents the geographical heterogeneity in the ownership and use of bed nets. Among a total of 14 states/regions and 1 union territory, 13 exhibited high ownership rates (> 96%) of any type of bed net, whether conventional or ITN. Only two regions, Kayin (87.3%) in the south-eastern and Shan (86.6%) in the east, reported ownership levels below 90%. Spatial analysis indicated a relatively noncoherent distribution in the regions of Chin (57.0%), Kayah (58.8%), Shan (62.9%), and Kayin (68.0%).

Overall ITN ownership was lowest in the central part of the country, including Yangon (4.5%), Bago (9.1%), Ayeyarwady (11.6%), Mandalay (8.3%), and Nay Pyi Taw (4.8%). By contrast, the highest rates of ITN ownership were observed in the western (Chin: 71.2% and Rakhine: 53.8%), south-eastern (Kayah: 76.1%), and southern (Mon: 56.1% and Tanintharyi: 65.8%) regions. There was a highly homogeneous distribution between access to and use of ITNs within almost all states/regions. The use of ITNs was highest in Tanintharyi (41.3%), Chin (40.0%), and Kayah (39.8%). The lowest use of ITNs was found in Nay Pyi Taw (3.2%), Yangon (3.8%), and Bago (5.5%) (Fig. 2).

### Correlation between ownership and use of bed nets

Table 3 presents the correlation between ownership and the use of bed nets. The Pearson correlation coefficient indicates a statistically significant relationship between access to ITNs and their actual use on the night before the survey (r: 0.9795, 95% CI 0.9377, 0.9933, alpha < 0.001). However, no correlation was observed between ownership of any net and the use of any net.

**Table 3:** Correlations between ownership and use of bed nets

	Slept under any net last night	Slept under ITN last night
Own any net	0.5127 (0.0006, 0.8118)^a^	–
Access to ITN	–	0.9795 (0.9377, 0.9933)^a^*

^*^Significance at alpha < 0.001; ^a^ r value and 95% confidence interval according to the Pearson correlation coefficient

## Discussion

The malaria transmission cycle takes place when the parasite, vector, and their environment converge [26]. To break the cycle, individuals residing in malaria-prone areas must adopt appropriate preventive measures. The use of ITNs has been proven effective in preventing malaria infection, minimising adverse outcomes after infection, and interrupting onward transmission within a community [9, 14]. However, persistently low rates of ITN usage have been reported, particularly in specific population groups, such as migrant workers in remote forest settings [11, 13, 15, 18, 27]. Likewise, in this study, despite high rates of net ownership and the use of any type of net, access to and use of ITNs remain relatively rare. The observed synergy between ownership and ITN use, indicates the need to expedite the distribution of ITNs while also promoting their use [19, 28]. The implementation of a microstratification plan detailing the malaria situation and related corresponding distribution strategies is essential for maximising the coverage of preventive measures in populations at risk of malaria.

This study found that the use of ITNs was highest in coastal regions among all areas, including delta and lowland regions, hills, and plains, aligning with the findings of some other studies [16, 25]. Delta and lowland areas, predominantly made up of states/regions in central Myanmar, have a lower malaria burden than other regions [4, 5, 8]. Further, the life cycle of the malaria parasite in *Anopheline* mosquitoes requires specific environmental conditions, including certain temperatures [29]. For instance, the sporogonic cycle cannot occur when the temperature falls below 20 °C (68° F) [30]. For this reason, malaria is seldom reported in certain hilly areas that have lower temperatures. This may contribute to a general lack of awareness concerning ITN use. Regardless of the malaria burden and the particular strategies used for the distribution of ITNs at each location, the ownership of any type of net was consistently high across the country. However, access to ITNs has remained relatively poor, resulting in very low usage rates for ITNs in all states/regions. Notably, some regions have a high prevalence of malaria, such as Tanintharyi and Rakhine in the coastal area [31, 32]. These areas are focal points for the NMCP that implement intensive malaria control activities, including biennial ITN distribution. However, earlier studies in Rakhine and Tanintharyi have indicated poor ITN utilization among specific population groups, such as migrant workers, fishers, and rubber tappers [18, 33]. These cohorts have high mobility and typically lack exposure to conventional ITN usage, leading to the need for additional resources, such as forest-adaptable hammock nets. In spite of this, investigations conducted in Myanmar and other countries in Southeast Asia indicate that both the adoption of personal protective measures and the utilisation of hammock nets remain limited [12, 34–36]. Future studies in all states and regions should explore what factors hinder or prevent the use of ITNs in different demographic groups.

Myanmar exhibits significant disparities between its urban and rural areas in terms of infrastructure, including health facilities, transportation, and the environment [37]. Major malaria vectors are predominantly found in rural areas, where favourable environmental conditions exist. Thus, in Myanmar, reports of malaria cases are more prevalent in rural regions than in urban ones [38]. This suggests that individuals who are residing in rural areas may have a greater awareness of the risks associated with malaria than their urban counterparts. Malaria projects are intended to provide intensive control activities, including health education interventions that primarily target rural areas where malaria is endemic. This may contribute to an overall increase in knowledge, attitudes, and practices in these communities with respect to malaria prevention [39]. As a result, individuals in rural areas are more likely to use ITNs, which aligns with findings from similar studies also conducted in Myanmar [22, 25]. However, it is crucial to consider a different strategy for net distribution for the NMCP, to strike a balance between the ownership and usage of ITNs. This strategy, which relies solely on malaria incidence, led to the distribution of the most ITNs in rural areas, producing high ownership and subsequently higher usage. The higher rate of non-use among the urban population may be attributable to a lack of access to ITNs. Thus, even in central regions, such as Yangon, residents of certain peripheral townships remain at risk of malaria infection [5]. Future studies should be conducted at least at the township level, producing more granular outcomes and adjusting the balance between distribution and usage. At present, the Myanmar NMCP distributes deltamethrin-treated polyester nets. While rural areas show high ownership and usage of ITN, challenges such as insecticide resistance and early or outdoor vector biting persist [40, 41]. Moreover, Myanmar’s proximity to countries like India, where the urban malaria vector *Anopheles stephensi* is prevalent, poses a significant risk of the spread of malaria [42].

In this study, wealth quintiles were assessed based on various factors, including individual ownership of assets and income. Malaria predominantly affects vulnerable populations and is often linked to poor socioeconomic conditions [43]. The structure of the home can serve as a proxy for the risk of transmission [38]. Typically, affluent individuals reside in well-constructed brick houses that facilitate the installation of window screens that are a deterrent to the entry of mosquitoes. Such affluent individuals may therefore encounter a lower mosquito density and, overconfident in their protection, may not prioritize the use of ITNs. Conversely, individuals at a lower economic status may be concerned about falling ill. Due to the universal healthcare coverage being below the target, the prospect of spending one’s savings on potential treatments or being unable to work due to illness becomes a significant concern [44]. Either challenge could reduce total family incomes. Additionally, in Myanmar, regardless of the provision of free malaria diagnosis and treatment by basic health staff and trained village health volunteers, individuals often seek treatment from unauthorised practitioners [45, 46]. Those with the lowest wealth quintiles may be apprehensive concerning the cost of malaria treatment. Therefore, individuals with lower wealth quintiles are more likely to adopt ITNs as a preventive measure. This finding aligns with the findings of other studies that indicate that ITN usage tends to be lower among individuals with greater wealth [16, 25]. Consequently, efforts should be intensified to promote the use of ITNs among individuals with higher wealth quintiles, especially those living in malaria-endemic areas.

This study has both strengths and limitations. Notably, it is the first report to document the ownership and use of ITNs among the general population, utilising a nationally representative survey dataset. The substantial size of the sample used in this study enhances the generalisability and representativeness of the results. However, it is essential to acknowledge that the data, collected in 2015–2016, may not reflect the current situation in Myanmar precisely, given the ongoing challenges that are posed by the COVID-19 pandemic and recent political unrest. These factors significantly impede malaria control. Nevertheless, the findings of this study may provide valuable baseline data for informing future implementation activities to guide further research. A limitation of the study lies in its sole reliance on questionnaire-based data collection, which may not accurately capture the actual ownership and use of ITNs, along with other variables, such as access to mass media, among the younger population. To provide a more comprehensive understanding, it is recommended to incorporate direct observations of the actual presence and use of ITNs where feasible in future research. Additionally, the use of secondary data imposes limitations on the availability of variables to be included in the analysis. For instance, the chosen outcome variable in this study was use of ITNs on the night before the survey. This approach may categorise individuals who use ITNs every day except the previous night to the survey as non-users. Moreover, crucial variables potentially linked to the use of ITNs, such as occupational type and migration status, were not available. Hence, it is recommended that these factors be incorporated in future studies. Recognising the importance of consistent ITN use for effective malaria prevention, future studies should consider a more nuanced approach to data collection.

## Conclusions

In pursuit of achieving nationwide malaria elimination by 2030, it is imperative to implement intensive measures, especially through reinforcing malaria prevention with the widespread use of ITNs among high-risk individuals. However, the present study identifies a significant shortfall in the overall access and use of ITNs. To address this, there is a critical need to enhance the distribution of ITNs, with a specific focus on individuals residing in high-risk malaria states/regions, including Rakhine and Tanintharyi, by employing a precise microstratification strategy. This requires an efficient supply chain, given the observed correlation between access to and use of ITNs. Despite expanding access, efforts should be directed toward bolstering the actual usage of ITNs. Targeted health promotion interventions are particularly essential for individuals with relatively higher socioeconomic status residing in delta and lowland regions, hills, and plains. To further improve the effectiveness of vector control, it is essential to delve deeper into the underlying reasons hindering the use of ITNs or the preference for other types of vector control within the community, possibly through qualitative research methods.

## Data Availability

All the data that support the findings of this study are included in the manuscript. For those interested in accessing the raw data, a request can be submitted to the DHS Programme (https://dhsprogram.com/).

## Abbreviations

aOR: Adjusted odds ratio
cOR: Crude odds ratio
ITN: Insecticide-treated net
IRS: Indoor residual spraying
LLIN: Long-lasting insecticidal net
MDHS: Myanmar Demographic Health Survey
MoH: Ministry of Health
NMCP: National Malaria Control Programme
WHO: World Health Organization
95% CI: 95% Confidence interval
